# Glycogen synthase is required for heat shock-mediated autophagy induction in neuronal cells

**DOI:** 10.1242/bio.061605

**Published:** 2025-02-17

**Authors:** Pratibha Bhadauriya, Akanksha Onkar, Kamali Nagarajan, Kavikumar Angamuthu Karuppusamy, Subramaniam Ganesh, Saloni Agarwal

**Affiliations:** ^1^ Department of Biological Sciences and Bioengineering, Indian Institute of Technology, Kanpur 208016, India; ^2^ The Mehta Family Centre for Engineering in Medicine, Indian Institute of Technology, Kanpur 208016, India; ^3^ Gangwal School of Medical Sciences and Technology, Indian Institute of Technology, Kanpur 208016, India

**Keywords:** Stress response, Neuronal cells, Misfolded proteins, Metabolism, Proteolysis

## Abstract

Autophagy is an essential cellular process that facilitates the degradation of aggregated proteins and damaged organelles to maintain cellular homeostasis and promote cell survival. Recent studies have indicated a direct role for glycogen synthase (GS) in activating neuronal autophagy and in conferring protection against cytotoxic misfolded proteins. Since heat shock induces protein misfolding and autophagy is an essential component of the heat shock response that clears the misfolded proteins, we looked at the possible role of GS in heat shock response pathways in neuronal cells. We demonstrate an increase in the activity and level of GS and a concomitant increase in the glycogen level during the heat shock and post-heat shock recovery period. These changes had a direct correlation with autophagy induction. We further demonstrate that heat shock transcription factor 1 regulates the level and activation of GS during heat shock and that GS is essential for the induction of autophagy during heat stress in neuronal cells. Intriguingly, the partial knock-down of GS led to increased death due to heat shock in neuronal cells and *Drosophila*. Our study offers a novel insight into the role of GS and glycogen metabolic pathways in heat shock response in neuronal cells.

## Teaser

**Summary:** This study shows that heat shock increases glycogen synthase (GS) activity and levels. This study also shows that HSF1 regulates GS activation, and that GS is necessary for autophagy induction and cell survival during heat shock stress.

## INTRODUCTION

Various environmental stressors, including heat stress, oxidative stress, hypoxia, and DNA damage, induce the accumulation of misfolded and unfolded proteins both *in vitro* and *in vivo* (Wang and Kaufman, 2016). The ability to endure and thrive in adverse circumstances necessitates a delicate balance between the process of protein refolding and degradation, which is facilitated by a complex interplay of various regulatory proteins associated with cellular stress response pathways. An example of a regulatory mechanism involved in protein folding, refolding, and degradation of misfolded proteins is the heat shock factor-1 (HSF1)-mediated heat shock protein (HSP) chaperone system (Hartl et al., 2011). Moreover, the degradation of misfolded proteins is facilitated by various cellular processes, such as the ubiquitin-proteasome system (Löw, 2011), autophagy (Mizushima et al., 2008), and other mechanisms that rely on lysosomes (Cuervo and Wong, 2014). Autophagy has been demonstrated to be upregulated in various *in vitro* models under heat stress conditions (Jain et al., 2017; Nivon et al., 2009; Dokladny et al., 2013). Interestingly, there is a direct association between the activation of autophagy and HSF-1. It has been observed that changes in the activity of HSF-1 can impact the levels of LC3, a protein associated with autophagosomes (Zhao et al., 2009). This suggests a direct connection between HSF-1, the pathway responsible for the heat shock response, and autophagy (Dokladny et al., 2013).

The process of autophagy induction in response to heat stress encompasses multiple pathways. One proposed mechanism suggests that the accumulation of dysfunctional mitochondria occurs, resulting in a transition towards glycolytic metabolism and subsequently causing a decrease in overall ATP levels. The energy crisis results in about the activation of AMPK and the inhibition of mTOR, thereby initiating the process of autophagy (Jiang et al., 2019). The induction of autophagy is significantly influenced by the presence of reactive oxygen species (ROS) that are produced during heat stress. These ROS serve as secondary messengers, effectively stimulating the pathways associated with autophagy. ROS have been shown to facilitate the process of autophagy by inducing the oxidation of ATG4, a protein essential for the formation of autophagosomes. Additionally, ROS can inhibit the activity of mTOR, a key regulator of autophagy, in a manner that is dependent on AMPK (Alexander et al., 2010; Scherz-Shouval et al., 2007). HSF1 plays a role in regulating autophagy by directly regulating the expression of the autophagy-related gene *ATG7* (Desai et al., 2013). Furthermore, it has been observed that HSF1 can directly trigger autophagy, as demonstrated in the model organism *C. elegans* (Kumsta et al., 2017). Nevertheless, further study is required to investigate the regulatory mechanisms of autophagy under stressful circumstances. The activity of HSF1 has been observed to increase in response to oxidative and proteotoxic stress. Furthermore, a prior investigation conducted by our research group has demonstrated that under conditions of oxidative and proteotoxic stress, there is an upregulation of glycogen synthase activity, which plays a crucial role in inducing the autophagic flux (Rai et al., 2018). The regulation of autophagy by HSF-1 has been attributed to various mechanisms, but the specific molecular pathway through which this control occurs, particularly in the context of neuronal heat stress, remains largely unexplored.

Several recent studies have elucidated the involvement of glycogen synthase (GS) in the modulation of autophagy and cellular viability under various physiological stress circumstances (Rai et al., 2018; Onkar et al., 2020, 2023; Sheshadri et al., 2021). Notably, glycogen accumulation has been reported during heat shock (Moon et al., 2003) and GS has been found to induce autophagy in neurons exposed to oxidative stress and proteotoxic stress (Rai et al., 2018). Nevertheless, there is a lack of studies correlating glycogen accumulation, GS, and the initiation of autophagy during periods of heat stress and the subsequent recovery phase. It is of interest to note that the hypoxia-inducible factor-1 alpha (HIF-1α), one of the upstream activators of HSF1 (Agarwal and Ganesh, 2020), regulates GS activation under hypoxia (Pescador et al., 2010). Our current investigation aims to provide a comprehensive understanding of the role played by glycogen and GS in the heat shock response pathway and cell survival. Using neuronal cell lines and the *Drosophila* model, we demonstrate here that activation of GS confers protection to neurons during heat shock by inducing autophagy.

## RESULTS

### Heat shock induces the activation of GS and a concomitant increase in GS in neuronal cells

**Fig. 1.**
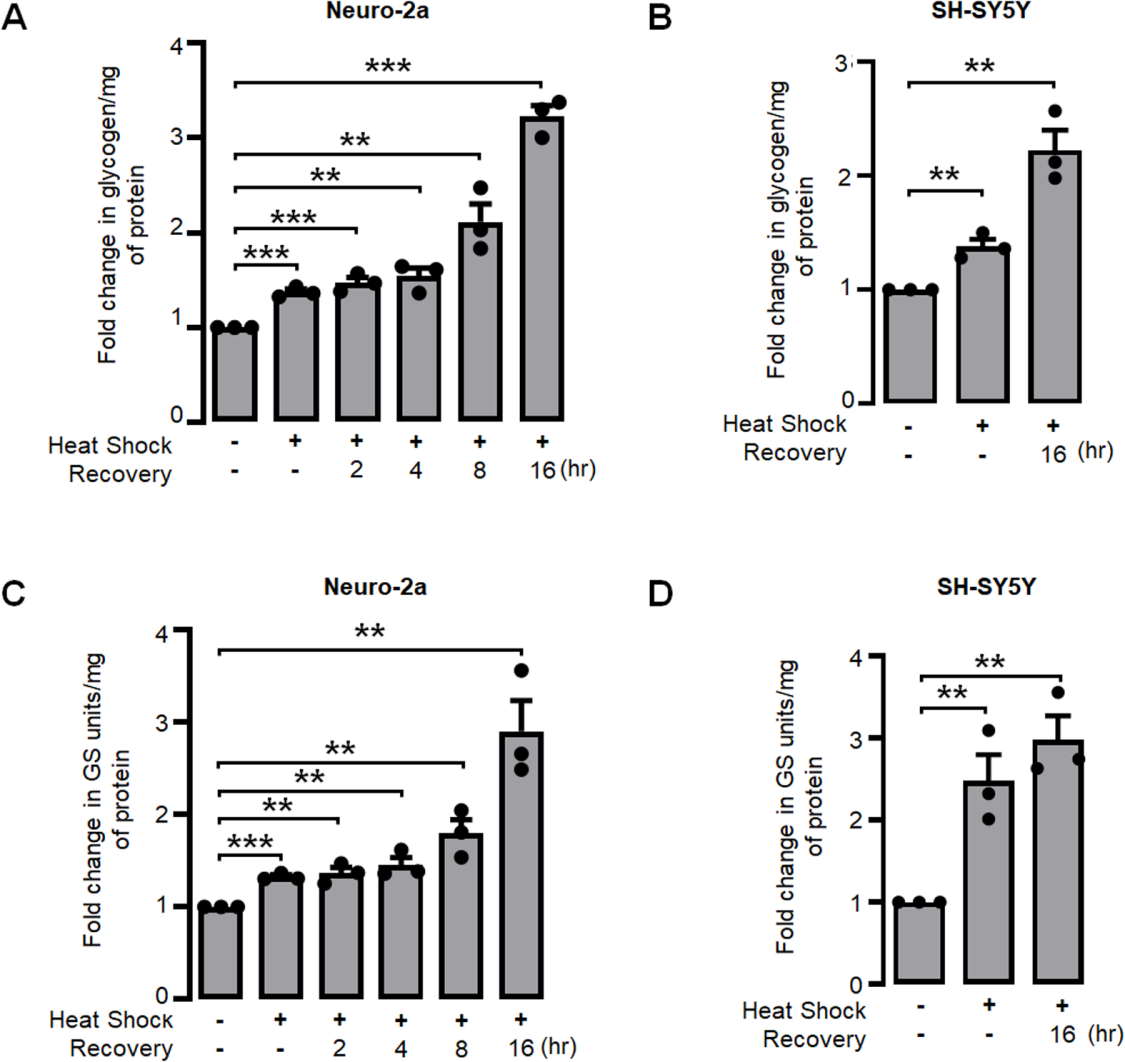
**Heat shock increases glycogen levels and activates GS in neuronal cell lines**. Bar diagram showing the fold change in glycogen levels during control, heat shock, and recovery periods in (A) Neuro-2a and (B) SH-SY5Y cell lines. Bar diagram depicting the fold change in overall GS enzyme activity during control, heat shock, and recovery periods in (C) Neuro-2a and (D) SH-SY5Y cell lines. Values for both GS activity and glycogen levels were normalized to the total protein content. Each bar represent mean±s.e.; *n*=3; **P<0.01; ***P<0.001.

Several studies have demonstrated an increase in glycogen levels in neuronal cell lines when exposed to physiological stresses, including endoplasmic reticulum (ER) stress and hypoxia (Wang et al., 2013; Saez et al., 2014; Rai et al., 2018). The present study proposed that the synthesis of glycogen in neuronal cells could be a physiological reaction to heat stress. Therefore, we examined the levels of glycogen in neuronal cell lines subjected to heat stress. For this, the Neuro-2a cells (a cell line derived from mouse neuroblastoma) and the SH-SY5Y cells (human neuroblastoma cell line from a metastatic bone tumor) were subjected to a heat shock at 42°C for 1 h. Subsequently, the cells were returned to 37°C to facilitate recovery and were harvested at specified time intervals. A significant increase in glycogen levels was observed during the periods of heat shock and subsequent recovery in comparison to the control condition (Fig. 1A). Similarly, it has been noted that there was a significant increase in glycogen levels in the SH-SY5Y human neuroblastoma cell line following exposure to heat shock and a subsequent 16-h recovery period (Fig. 1B), compared to the control condition. We next evaluated the enzymatic activity of GS in both Neuro-2a and SH-SY5Y cells under heat shock and subsequent recovery periods. For this, we assessed the activity of the GS enzyme. Consistent with expectations, a direct correlation was observed between GS activity and glycogen levels. Specifically, GS activity increased during heat stress and recovery phases (Fig. 1C). A similar observation was made for the SH-SY5Y cell line (Fig. 1D). Therefore, the aforementioned observations indicate that heat stress induces GS activity and leads to glycogen accumulation in neuronal cells. Subsequent investigations were conducted at the 16-h recovery time point, as it was determined that the maximum glycogen accumulation occurred at this specific interval. Further, all subsequent experiments were conducted only in Neuro-2a, a cellular model that has been extensively used for similar studies (Jain et al., 2017; Upadhyay et al., 2017; Rai et al., 2018; Bhadauriya et al., 2021).

### Heat shock-induced increase in the activity of GS correlates with an increase in GS protein levels and autophagy induction in neuronal cells

**Fig. 2.**
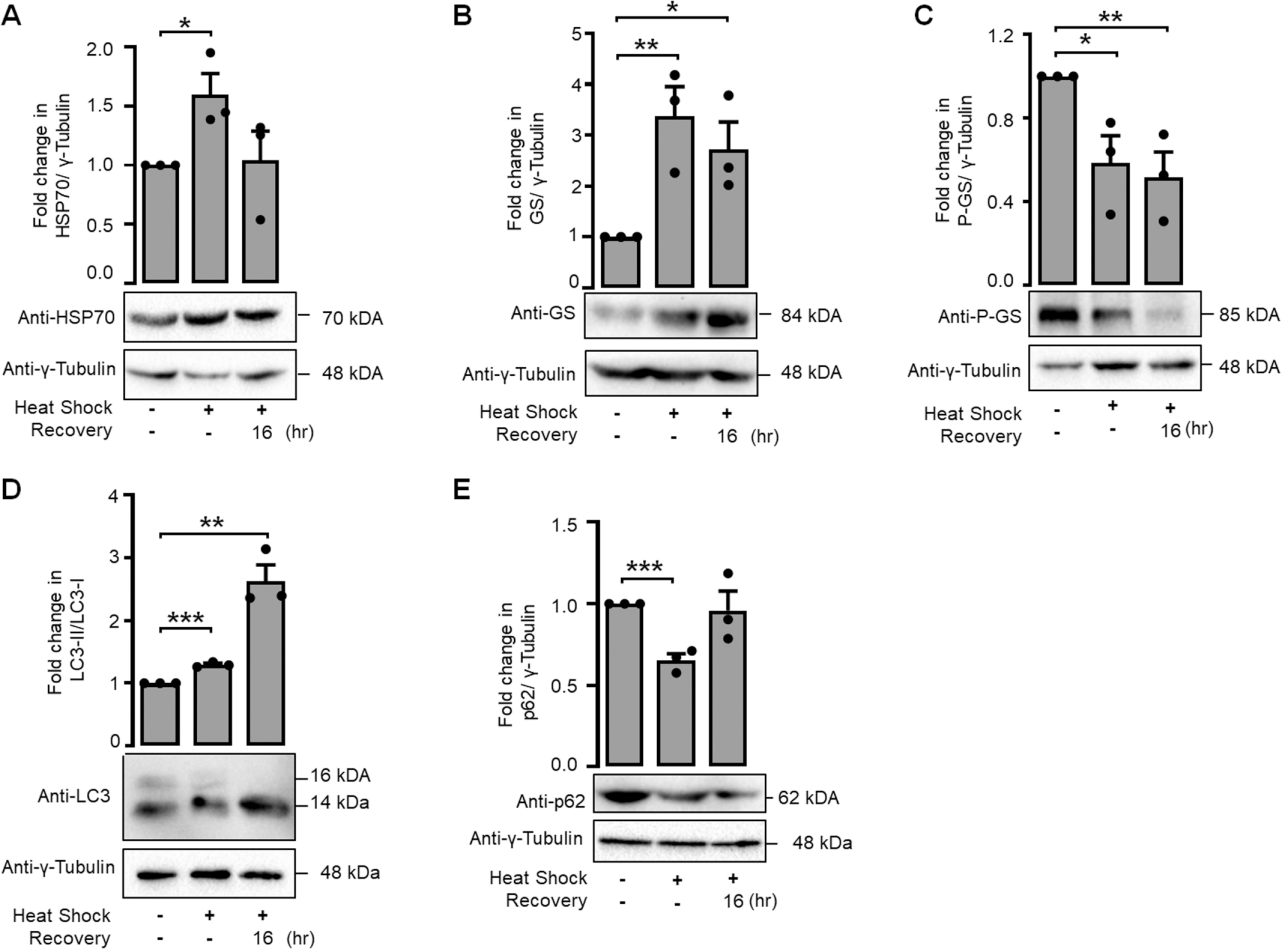
**Heat shock upregulates GS protein levels with a parallel induction in autophagy.** Representative immunoblots (lower panel) and bar diagrams (upper panel) showing the relative levels of total HSP70 (A), GS (B) and its phospho-S641 form (C), p62(D) and LC3II/I (E) in Neuro-2a cells during control, heat shock, and recovery periods. Protein levels of HSP70 served as the positive control for heat shock induction. Values for HSP70, GS and P-GS and p62 were normalized to γ-tubulin, which served as the loading control. Data shown in B,C,D,F,G, and I are represented as mean±s.e.; (*n*=3); *P<0.05; **P<0.01; ***P<0.001; ****P<0.0001.

After observing an elevation in glycogen levels and GS activity following heat shock and subsequent recovery, we wanted to investigate whether the activation of GS occurred at the protein level. Because the upregulation of HSP70 protein levels has been established during heat shock treatment (Kregel, 2002), we measured the HSP70 protein levels as a positive control readout of heat shock response. As expected, an increase in the HSP70 level was observed in the cells exposed to heat shock (Fig. 2A). Similarly, a notable increase in GS protein levels was observed during heat shock and in the subsequent recovery phase (Fig. 2B). We also examined if the increase in the GS level correlates with the decrease in the level of its inactive form, specifically the phospho-Ser641 GS (Marr et al., 2022). We found a significant reduction in the levels of phospho-Ser641 GS protein following heat shock and the subsequent recovery period (Fig. 2C). These observations indicate an upregulation of glycogen and GS protein levels and activity during heat shock.

**Fig. 3.**
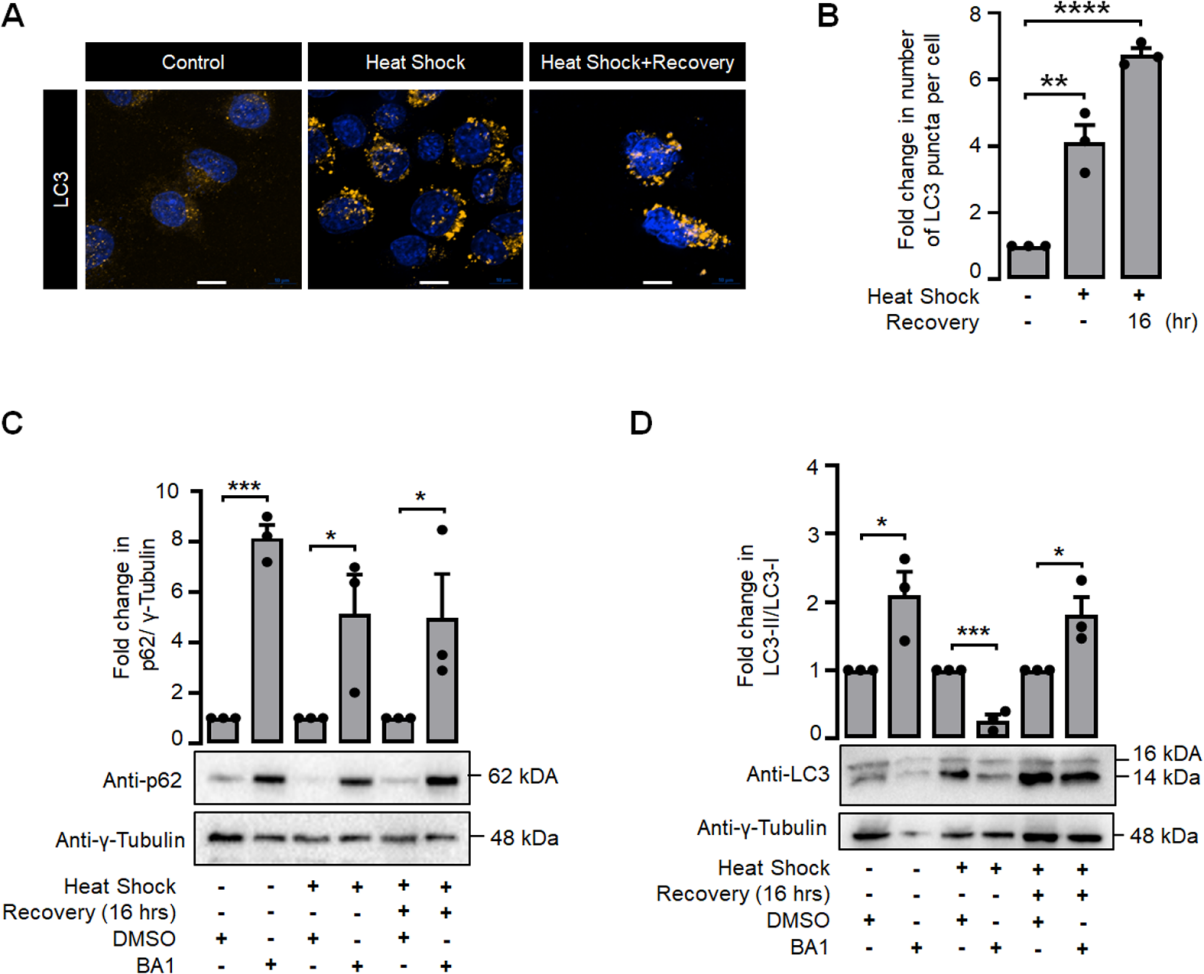
**Autophagy flux was affected during heat stress.** Representative immunofluorescence images (A) showing the endogenous level of total LC3 protein (yellow puncta) and bar diagram (B) showing the fold change in the number of LC3 puncta per cell in control, heat shock and recovery periods (35-40 cells each set). The nuclei in were stained with DAPI (scale bars: 10 µm). Representative immunoblot (lower panel) and bar diagram (upper panel) showing the p62 protein levels (C) and LC3-II/LC3-I (D) during control, heat shock and recovery phases in combination with BA1 treatment. γ-tubulin served as the loading control. Data shown in B,C and D are represented as mean±s.e.; (*n*=3); *P<0.05; **P<0.01; ***P<0.001; ****P<0.0001.

Heat shock is known to induce autophagy (Jain et al., 2017), and GS is known to induce autophagy in neuronal cells (Rai et al., 2018). To check if the activation of GS and the increase in the glycogen content during heat shock correlate with autophagy induction, we examined the protein levels of p62 and the ratio of LC3-II to LC3-I during both heat shock and the subsequent recovery period. A significant decrease in p62 protein levels was observed during heat shock, as shown in Fig. 2E. Moreover, the LC3- II/LC3-I protein levels significantly increased during both the heat shock and recovery phases, as shown in Fig. 2F. This observation indicates a substantial increase in the upregulation of autophagy during heat shock and recovery phases. We further confirmed this by immunostaining autophagosomes with an antibody for LC3 and quantifying the puncta. As shown in Fig. 3A and B, there was a significant increase in the number of LC3-positive puncta in cells exposed to heat shock and at 16 h post-heat shock recovery period. To assess autophagy flux, Neuro-2a cells were treated with 10 nM bafilomycin A1 (BA1) for 24 h. Following treatment, the cells were washed to eliminate BA1-containing media and underwent heat shock. Fig. 3C shows a significant elevation in p62 protein levels subsequent to BA1 treatment, thereby corroborating the induction of autophagy inhibition by BA1 under both control and heat shock conditions, as well as during the recovery phases. The autophagy flux was additionally confirmed by assessing the ratio of LC3-II to LC3-I protein levels during the heat shock and recovery phases. Fig. 3D illustrates a significant increase in the LC3-II/LC3-I protein ratio in the control and recovery phases post-BA1 treatment, signifying enhanced autophagy flux during recovery. Conversely, the LC3-II/LC3-I ratio diminished promptly following heat shock, while p62 levels increased during an autophagosome blockade. We additionally quantified LC3 puncta during the control, heat shock, and recovery phases, with and without BA1 treatment. Fig. S1A illustrates that the quantity of LC3 puncta was markedly elevated in BA1-treated cells under control conditions, signifying enhanced autophagy flux. Nonetheless, no substantial difference was noted during the recovery phase. Collectively, these observations indicate that autophagy flux is elevated during the recovery phases.

### Heat-shock-induced increase in the level and activity of GS is a survival mechanism during stress

The increases in LC3-positive puncta correlate well with the increase in GS level, activity, and glycogen levels, confirming a causal role for GS in autophagy induction.

**Fig. 4.**
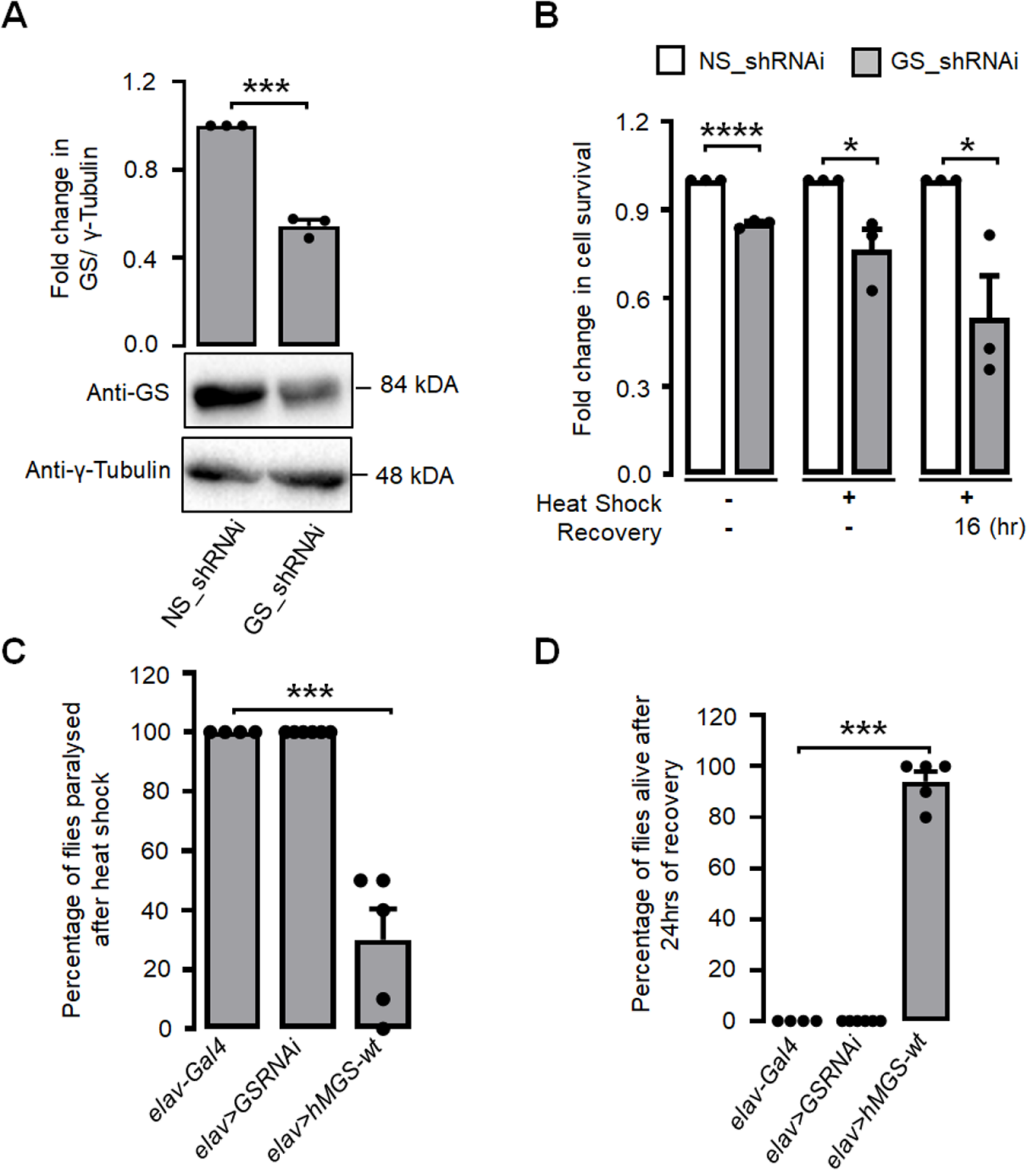
**Glycogen accumulation and GS activation plays a protective role during heat stress.** (A) Bar diagram (top panel) and representative immunoblot (bottom panel) depicting the fold change in total GS levels in control plasmid transfected (NS_shRNAi) and GS_shRNAi transfected Neuro-2a cell lines. γ-tubulin served as the loading control. (B) Bar diagram representing the fold change in cell survival in NS_shRNAi and GS_shRNAi transfected Neuro-2a cell lines during control, heat shock, and in 16 h. recovery period as indicated. Bar diagram comparing the percentage of flies paralyzed upon heat shock treatment (C) and percentage alive after 24 h of recovery (D) in control (elav-Gal4), GS-knocked down (elav>GSRNAi) and GS-overexpressing (elav>hMGS-wt) groups. Each bar represents mean±s.e.; *n*=3; *P<0.05; ***P<0.001; ****P<0.0001.

Given the observed upregulation of both the level and activity of GS in neuronal cells during the heat shock and subsequent recovery phase, our objective was to investigate the potential dependence of GS in facilitating an efficient response by neuronal cells to heat shock. In this study, we employed the RNA interference (RNAi) technique to partially suppress the expression of GS (Fig. 4A). Subsequently, we examined the impact of this gene silencing on survival rates under conditions of heat shock. As shown in Fig. 4B, it was observed that Neuro-2A cells, which were transfected with the RNAi construct targeting the GS gene, exhibited a notable reduction in cell viability following a heat shock event or during the subsequent recovery period. In order to enhance the credibility of these results in an *in vivo* setting, we have employed a *Drosophila* model in which the native GS is partially suppressed *(elav>GSRNAi)*, or the human GS was overexpressed in the brain *(elav>hMGS-wt)*. As shown in Fig. 4C, a significant number of flies overexpressing the human GS did not experience paralysis following heat shock, in contrast to the majority of wild-type flies and flies with partial GS silencing. In a comparable way, the proportion of flies that remained alive after a 24-h period following a heat shock event was notably greater in the transgenic flies that overexpressed GS in comparison to the wild-type flies or the flies with a partial suppression of GS (Fig. 4D). These observations implies that the GS gene serves a protective function in the context of heat shock.

**Fig. 5.**
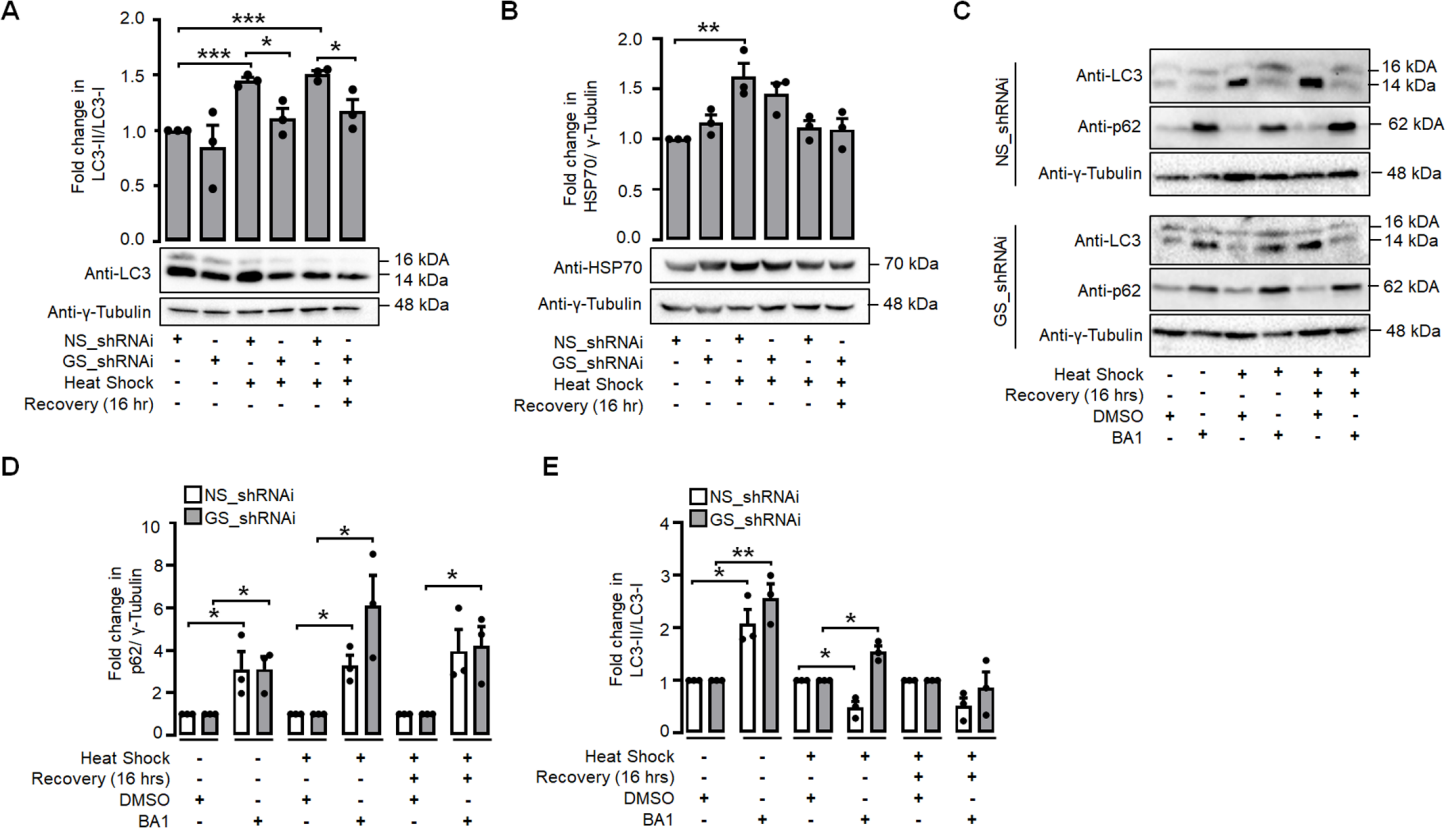
**GS regulates autophagy induction during heat shock.** Top panel: bar diagram showing fold change in LC3II/I (A), and HSP70 protein levels (B); bottom panel, representative immunoblot showing the protein levels of LC3II/I (A) and HSP70 protein levels (B) in NS_shRNAi and GS_shRNAi-transfected Neuro-2a cell lines during control, heat shock and 16 h, recovery as indicated. γ-tubulin serves as the loading control. Representative immunoblot (C) and bar diagram illustrate the p62 (D) and LC3 (E) protein levels in Neuro-2a cell lines transfected with NS_shRNAi and GS_shRNAi, treated with DMSO or BA1, during the heat shock and recovery phases as indicated. Data shown represents mean±s.e.; *n*=3; *P<0.05; **P<0.01; ***P<0.001.

Given the observed positive correlation between the GS level and the induction of autophagy during heat shock and considering the established role of GS in regulating autophagy (Rai et al., 2018), our next objective was to investigate the requirement of GS in heat shock-induced autophagy in the neuronal cells. For this, Neuro-2a cells were subjected to transient transfection with the RNAi construct targeting the GS transcripts. Subsequently, the cells were subjected to a heat shock treatment, and the induction of autophagy was assessed by measuring the levels of LC3. This assessment was performed either immediately after the heat shock or 16 h after the heat shock treatment. As shown in Fig. 5A, a significant decrease in LC3 levels was observed in cells that underwent partial gene silencing of GS and were subsequently subjected to heat shock. This finding implies a potential causal relationship between GS and the induction of autophagy in neuronal cells following heat shock. Additionally, we also tested if the absence of GS would have any detrimental effects on the heat shock response pathway. This is of particular interest because previous studies have demonstrated that heat shock stress response pathways play a role in regulating the autophagy process (Dokladny et al., 2013). For this, we quantified HSP70 protein levels in Neuro-2a cells upon partial inhibition of GS and subsequent exposure to a heat shock. As shown in Fig. 5B, there was no significant difference observed in the HSP70 levels among cells partially silenced for GS. This finding implies that the activation of HSF1 and its targets remain unaffected in the absence of GS. To confirm whether partial knockdown of GS affects autophagy flux, we quantified the p62 levels and LC3-II/LC3-I ratio in Neuro-2a cells transiently knocked down for GS. As shown in (Fig. 5C-E), the increased p62 and LC3-II/LC3-I ratio in GS knockdown cells under heat shock conditions with BA1 treatment indicates that GS inhibition further impairs autophagy flux during heat stress. This finding was further validated by quantifying p62 protein levels, which were consistent with the LC3-II/LC3-I ratio, suggesting that GS plays a regulatory role in autophagy during heat stress.

### GS levels are regulated by HSF1 during a heat shock

**Fig. 6.**
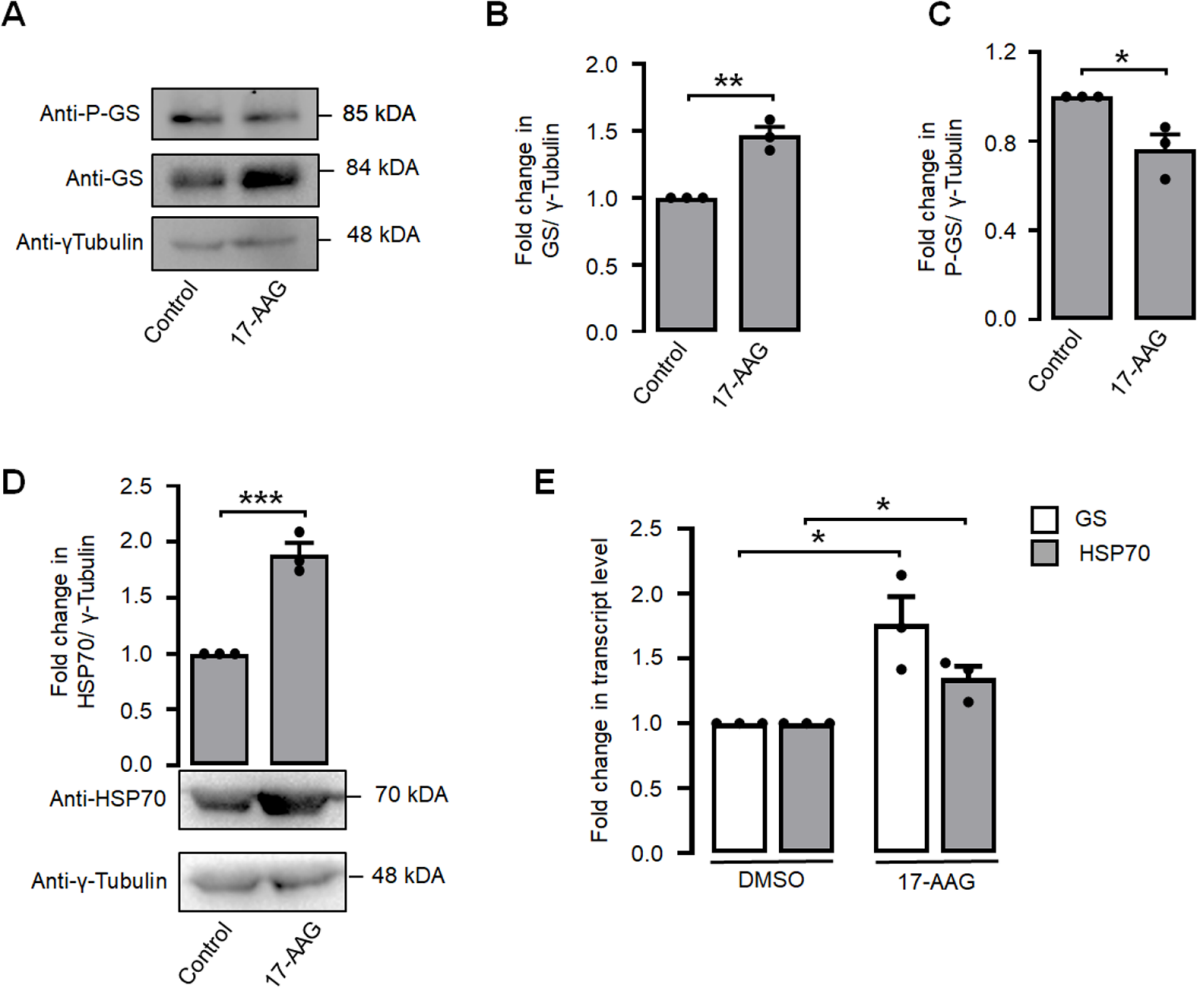
**17-AAG treatment upregulates GS in neuronal cells.** Representative immunoblot (A) and bar diagrams (B,C) showing the relative protein levels of total-GS and P-GS, in control and 17-AAG treated Neuro-2a cells. Values were normalized to γ-tubulin, which served as the loading control. (D) Image showing the fold change of HSP70 protein levels as depicted in the bar diagram (top panel) and immunoblot (bottom panel) in control versus 17-AAG treated Neuro-2a cells. (E) Bar diagram showing the relative levels of GS transcript in control versus 17-AAG treated cells. The transcript levels of the HSP70 gene served as the positive control for heat shock induction. Data are represented as mean±s.e.; *n*=3; *P<0.05; **P<0.01.

**Fig. 7.**
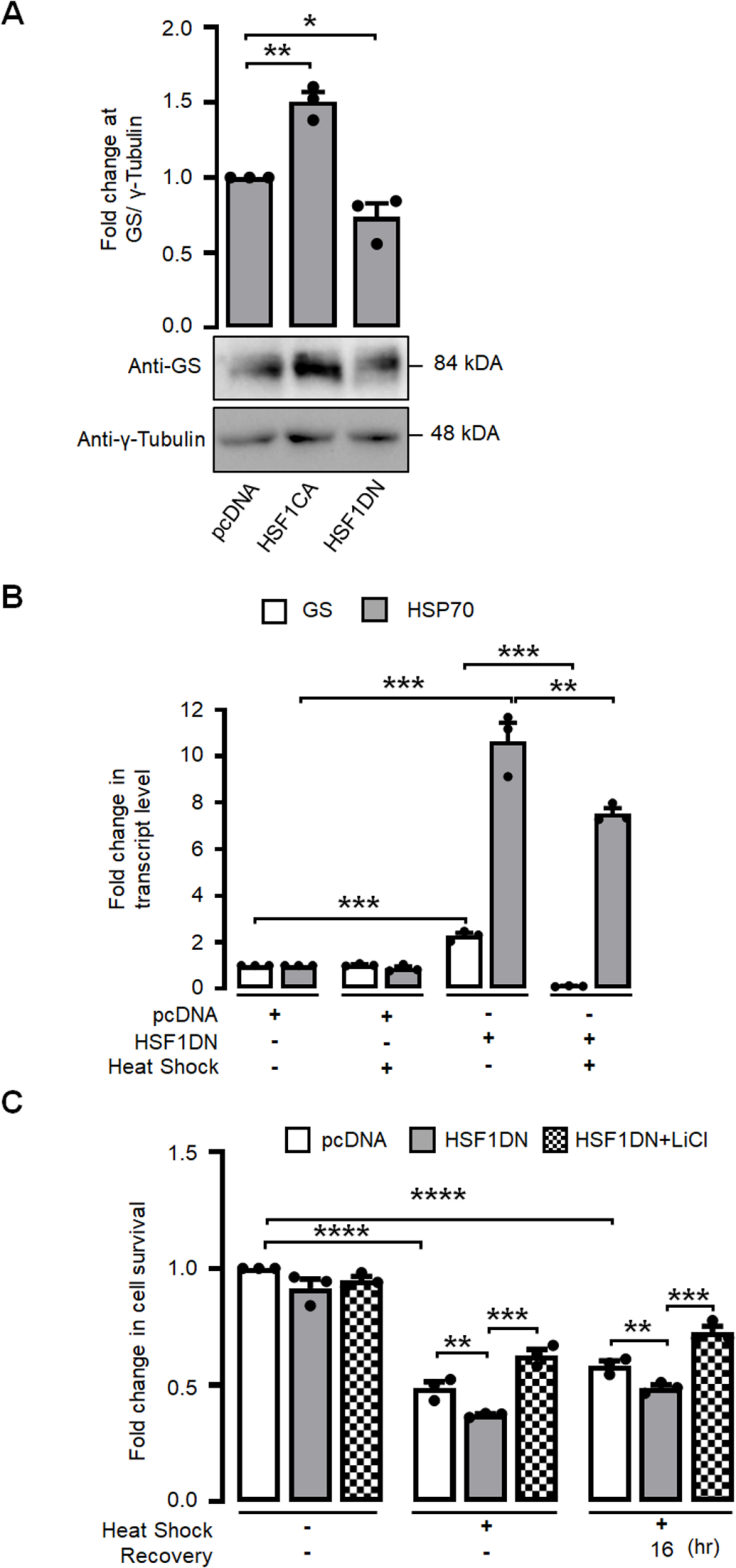
**HSF1 regulates GS activation during heat shock.** (A) Bar diagram (top panel) and immunoblot (bottom panel) showing the total GS protein levels in Neuro-2a cells transfected with pcDNA, HSF1CA, and HSF1DN plasmids as indicated. Values were normalized to γ-tubulin, which served as the loading control. (B) Bar diagram showing the fold change in HSP70 and GS transcript levels in Neuro-2a cells are transfected with either pcDNA or HSF1DN and subjected to heat shock as indicated. (C) Bar diagram showing the fold change in cell survival in control, heat shock, and during the recovery phase where the cell was transfected with pcDNA or HSF1DN plasmids and subjected to 10 mM LiCl treatment before heat shock as indicated. Data shown here are represented as mean±s.e.; *n*=3; *P<0.05; **P<0.01; ***P<0.001; ****P<0.0001.

The transcription factor HSF1 plays a pivotal role as the primary regulator of the cellular heat shock response. Consequently, our objective was to investigate the potential dependence of heat shock-induced activation of GS on HSF1. For this, we employed 17-AAG, an analogue of geldanamycin, to induce activation of the HSF1 in Neuro-2a cells. Subsequently, we quantified the levels of the inactive form of GS, specifically pGS-Ser641 (Sinadinos et al., 2014). The data presented in Fig. 6A, B, and C demonstrate that the administration of 17-AAG led to a significant decrease in the levels of the inactive variant of GS. Here, HSP70 protein level serves as the positive control (Schulte and Neckers, 1998) for 17-AAG treatment (Fig. 6D). To confirm our result, we measured the HSP70 and GS transcript levels during 17-AAG treatment and found that their transcript levels did increase significantly during the 17-AAG treatment (Fig. 6E). To further strengthen this observation, we transiently overexpressed a construct that codes for the constitutively active form of HSF1 or its dominant-negative variant in Neuro-2a cells, and then measured the levels of GS. We found that the expression of the constitutively active form of HSF1 resulted in a significant increase in the GS levels. Conversely, the expression of the dominant-negative form of HSF1 led to a reduction in GS levels, as shown in Fig. 7A. Further, to investigate whether HSF1 can regulate the GS transcript level during heat shock, we overexpressed the HSF1 dominant-negative construct and exposed the Neuro-2a cells to heat shock. As expected, during heat shock, there was a significant increase in the level of GS and HSP70 transcript (Fig. 7B). Conversely, there was a significant reduction in the level of GS and HSP70 transcript when HSF1 dominant-negative construct was overexpressed and exposed to a heat shock (Fig. 7B). This observation suggests that HSF1 is an upstream regulator of GS expression. To strengthen this notion, we carried out an *in-silico* analysis to identify potential HSF1 binding sites in the putative promoter region of GS gene, using the ConTra v3 tool (Kreft et al., 2017). We found that multiple potential HSF1 binding sites were present on the GS promoter region in diverse mammalian species and one of them was conserved in all the species studied (Fig. S2).

We next wanted to check if activation of GS could provide protection against heat stress in the absence of HSF1. In this experiment, the Neuro-2a cells were transfected with a construct coding for the dominant-negative HSF1 and the cells were subjected to treatment with lithium chloride, a well-established activator of GS. Following this, the cells were exposed to a heat shock, and their viability was assessed. As shown in Fig. 7C, cells that transiently overexpressing the dominant negative HSF1 exhibited decreased viability when exposed to heat stress, in comparison to the control group. Nevertheless, it was observed that cells subjected to lithium chloride treatment exhibited a significant enhancement in their survival rate, even when the activation of HSF1 was impaired. This finding implies that the GS plays a significant role in the neuronal cell survival pathways during a heat shock.

## DISCUSSION

Cellular protein quality control is the mechanism through which eukaryotic cells maintain the synthesis, functioning, and degradation of proteins to ensure their proper balance in normal conditions (Kim et al., 2023; Kampinga et al., 2019). During cellular stress, such as heat shock, hypoxia, ER stress, and proteotoxic stress, the proportion of misfolded proteins increases abnormally high and renders them aggregate-prone (Chen et al., 2011; Mittal and Ganesh, 2010). As a cytoprotective response, the cell activates a set of stress response pathways to reduce the negative effects of these misfolded proteins on the cell. The heat shock response mechanism is a significant pathway involved in maintaining protein homeostasis. This study shows that GS plays a key role in inducing autophagy during and after heat stress in neuronal cells.

Our investigation indicates that there is an increase in glycogen accumulation in neuronal cell lines during heat shock and subsequent recovery, which corresponds to the upregulation of GS activity. Further, we established the role of GS in inducing autophagy during heat stress and in the subsequent recovery stages in neuronal cells. Thus, our study establishes a novel role for GS in the survival of neurons during heat stress. Additionally, the accumulation of glycogen is also observed in other physiological stress, endoplasmic reticulum stress (Wang et al., 2013), including hypoxia (Pescador et al., 2010), and proteotoxic blockade (Rai et al., 2018; Onkar et al., 2023). The increase in GS activity and the improvement in autophagy flux that we observed during heat shock and the recovery phase are consistent with the discovery that GS is upregulated, and autophagy flux is enhanced during proteotoxic stress (Rai et al., 2018; Onkar et al., 2023). Defects in the autophagic process have been associated with several neurodegenerative disorders, and activation of autophagy was shown to ameliorate the disease phenotype in model systems (Guo et al., 2018). Given the possible role of HSF1 in the autophagic process (Dokladny et al., 2015) and our current findings on the role of GS in heat shock response, it is tempting to speculate a role for GS in stress response pathways in neurodegenerative disorders. Indeed, GS is shown to activate in the HD models (Rai et al., 2018), and the neuronal activation of GS was shown to ameliorate the degenerative phenotype in HD fly models, possibly by inducing autophagy (Onkar et al., 2023). Indeed, a functional link between GS and autophagy gene Atg8a has been reported in flies (Zirin et al., 2013), and the gene expression data analyzed in the present work further supports this notion. Thus, the GS-mediated survival process appears to be a conserved and generic stress response mechanism in neuronal cells.

Our findings indicate that GS is essential for cells to induce autophagy in response to heat stress, suggesting that the mortality observed in our study models (neuronal cell lines and flies) with partial silencing of GS is likely due to compromised autophagy during stress. The comparable levels of HSF1 and its target HSP70 in GS-silenced cells subjected to heat shock indicate that the primary role of GS during heat stress is the activation of autophagy, rather than the activation of HSF1 and/or the subsequent expression of HSPs. Therefore, GS seems to regulate only a subset of the heat shock response pathway, and HSF1 is one of the upstream regulators of GS during heat stress. This notion is strengthened by the observation that the GS protein was elevated in cells exposed to the HSF1 activator 17-AAG (Schulte and Neckers, 1998) or in cells overexpressed the constitutively active HSF1. Thus, HSF1 appears to function upstream of GS. Given that HSF1 functions as a transcription factor and that most players of the heat shock response pathways are controlled at the transcriptional level, it is plausible to propose that GS is among the direct targets of HSF1. Our bioinformatics analysis has identified potential binding sites for HSF1 in the upstream regions of the GS gene. Collectively, our findings strongly indicate that GS is a direct target of HSF1 and warrants further investigation.

Although the involvement of autophagy in the heat shock response has been confirmed (Dokladny et al., 2013; Latorraca et al., 2020; Kassis et al., 2021), the specific role of GS in neuronal cells during the recovery phase of heat shock remains uncertain. One plausible hypothesis is that glycogen serves as an energy reservoir for the recovery process (Roach et al., 2012). Another potential role of glycogen is to act as a crowding agent, preventing the aggregation of misfolded proteins (Rai et al., 2018; Onkar et al., 2023; Mittal and Singh, 2014) and aiding in their clearance. However, our discovery that the activation of glycogen-mediated autophagy continues during the recovery phase, even after 16 h following a heat shock, concurs with an independent report (Nivon et al., 2009). The authors proposed that increased autophagy during heat shock recovery is an important cellular response to the denaturation and aggregation of proteins caused by heat shock and is, therefore, crucial for cell survival. Clearly further studies are required to understand the molecular function of GS and glycogen in the heat response pathway and the other factors involved in the process.

## MATERIALS AND METHODS

### Reagents, antibodies, and expression construct

The following antibodies were used in the current study: anti-γ-tubulin (cat# T6557; Sigma-Aldrich India Pvt. Ltd.; dilution 1:10,000). Anti-phospho-GS (cat# 3891S; dilution 1:1000), anti-HSF1 (cat# 4356s; dilution 1:1000), anti-HSP70 (cat# 4872S; dilution 1:1000), anti-GS (cat# 3893; dilution 1:1000), anti-LC3II (cat# 27755S; dilution 1:1000) and anti-p62/SQSTM1 (cat# 5114S; dilution 1:1000) were purchased from Cell Signaling Technology Inc., USA. The secondary antibodies were obtained from Jackson ImmunoResearch Inc., USA. Thiazolyl Blue Tetrazolium Bromide (MTT), β NADH, uridine-5′-diphosphoglucose, D-glucose 6-phosphate, enzyme amyloglucosidase were purchased from Sigma-Aldrich Chemicals, New Delhi, India. Phospho-enol pyruvate was purchased from Thermo Fisher Scientific. Lithium chloride was purchased from Fine-Chem Ltd. The glucose estimation kit was purchased from ERBA Diagnostic Mannheim Gmbh LTD. The construct coding for GS knockdown, GS_RNAi, and overexpression of GS was reported in a previous study (Rai et al., 2018). The expression construct for dominant-negative HSF1 (HSF1Δ370-410) (Verma et al., 2014) was kindly provided by Professor Santosh R. D'Mello (Department of Molecular and Cell Biology, University of Texas at Dallas, USA). HSF1CA plasmid is a kind gift from Professor Akira Nakai (Department of Biochemistry and Molecular Biology, Yamaguchi University School of Medicine, Japan) (Hayashida et al., 2010).

### Cell culture, transfection, and treatment

Neuro-2a and SH-SY5Y cell lines were purchased from the National Centre of Cell Sciences (Pune, India) and grown in Dulbecco's modified Eagle's medium (GIBCO International) supplemented with 10% (v/v) fetal bovine serum (GIBCO International) and 1% antibiotic-antimycotic solution (Sigma-Aldrich). The cells were grown in aseptic incubators with 5% CO2, 37°C, and a humid environment. Cells were transfected with DNA constructs using Turbofect (Thermo Fisher Scientific, USA) transfection reagent at 60 to 70% confluency following the supplier's protocol. The transfected cells were subjected to treatment at 24 to 36 h post-transfection. Cells with 80-90% confluency were passaged to maintain running culture. For heat shock treatment, cells grown in 35 mm were treated at 42°C for 1 h in a humidified incubator with 5% CO_2_. For recovery from heat shock, cells were returned to the humidified incubator with 5% CO_2_ maintained at 37°C and harvested at the indicated time point. For inducing the GS activity, cells were treated with 10 mM LiCl for 1 h before heat shock.

### Glycogen estimation

The cellular glycogen content was quantified as described protocol (Rai et al., 2018), which involves measuring the release of glucose following the treatment of glycogen with the enzyme amyloglucosidase. Briefly, following the heat shock treatment, Neuro-2a and SH-SY5Y cells were suspended in 100 μl of 30% KOH buffer and subjected to boiling at 100°C for 20 min. 80 μl of the lysate was subsequently applied to Whatman paper 31-ET CHR filter paper, rinsed with 66% ethanol (three washes of 10 min each), and dried overnight at 37°C. Subsequent to drying, the filter paper was incubated for 1 h in 0.2 M sodium acetate (pH 4.8) with 0.5 mg/ml of amyloglucosidase. The glucose estimation kit was employed for the colorimetric assessment of glucose release from the filter paper. The estimated value was expressed as released glucose (μM) per milligram of protein. The protein concentration was determined utilizing the BCA method.

### GS enzyme activity

GS activity was estimated by modified Danforth's spectrophotometric method. Briefly, the reaction mixture consisted of 48 mM Tris (pH 8.2), 12.4 mM MgCl2, 1 mM EDTA, 2.4 mM 2-mercaptoethanol, 3.63 mM UDPG, 9.7 mM glucose 6-phosphate. Upon addition of enzyme collected from the test and control condition, an increase in absorbance at 340 nm was measured for a time interval of 5 min.

### Immunoblotting

The cultured cells were harvested and suspended in SDS-DTT lysis buffer (60 mM Tris-pH 6.8, 2% SDS, 100 mM DTT) to prepare cell lysate. The proteins in the cell lysates were resolved on SDS-PAGE, and the protein was transferred from the gel to a nitrocellulose membrane. The membranes were blocked for 1 h with 5% appropriate blocking buffer, either with bovine serum albumin (BSA) or non-fat dry milk powder dissolved in TBST (Tris-buffered saline, pH 7.6 and 0.1% Tween 20). The membrane was incubated with the primary and secondary antibodies using the manufacturer's protocol. The immunoreactive bands were then visualized using a chemiluminescence detection kit (SuperSignal West PICO Chemiluminescent Substrate, Thermo Fisher Scientific India Pvt. Ltd., Mumbai, India) and then imaged using a ChemiDoc imaging system (Bio-Rad Laboratories, India). Image analysis was performed by using Image Lab software version 5.2.1 (Bio-Rad Laboratories, India).

### Immunocytochemistry

For immunostaining, the cells were grown on a gelatin-coated glass coverslip and then harvested after the indicated treatment. Treated cells were fixed with 4% formaldehyde solution for 15 min. After the fixation cells were washed three times with 1XPBS for 5 min and then permeabilized with 0.05% Triton-X100 for 5 min as described previously (Agarwal and Ganesh, 2020). Subsequently, cells were washed three times with 1XPBS for 5 min and then incubated with blocking buffer (0.5% fish gelatin and 0.5% equine serum) for 1 h and then incubated with primary and secondary antibodies, dissolved in blocking buffer. Nuclei were visualized by staining the cells with DAPI (10 μM) for 5 min. Imaging was done by using a confocal microscope (Nikon Eclipse Ti2-E, Japan) at 60X magnification. Images were processed using NIS element AR software (Nikon Instruments Inc., Japan). Cell puncta were counted by using the ImageJ software.

### RNA isolation and quantitative real-time PCR

For RNA isolation, cells were harvested after the treatment as indicated. RNA was isolated by using the Trizol reagent as recommended by the manufacturer (Life Technologies India). Around 5 µg of RNA was used to synthesize the cDNA. Real time PCR was performed by using the GoTaq qPCR Master Mix (Promega Corporation, USA) on a CFX-96-real time PCR Machine from Bio-Rad Laboratories, India. For calculation GAPDH transcript levels were used to normalize the expression of the HSP70 and GYS1. The sequences of the primer pairs used in this study are given in Table S1.

### MTT Assay

For cell death assays, the Neuro-2a cells were seeded and grown in four-well plates. After transfection and treatment as indicated, the cells were incubated with 0.5 mg/ml of MTT dissolved in complete cell media and incubated at 37°C for 3 h. To aid dissolution of formazan crystals, the cells were incubated at 37°C for 20 min in DMSO. The absorbance was read at 565 nm using the Spectra-Max M3 Multi-Mode Microplate Reader (Molecular Devices).

### Transgenic fly stocks and culture and heat shock treatment

The pan-neuronal line *elav-Gal4^C155^* was a generous gift from Professor Pradip Sinha (IIT Kanpur, India). Transgenic fly lines for the wild-type form of hMGS (human Muscle Glycogen Synthase), *UAS-hMGS-wt* (wild-type form of the hMGS), or the *UAS-GSRNAi* (v35136) were generously provided by Professor Joan J. Guinovart (IRB, Barcelona, Spain). Males of the *UAS-hMGS-wt* and *UAS-GSRNAi* were crossed with the virgins of *elav-Gal4^C155^* to drive their pan-neuronal expression (*elav>GSRNAi* and *elav>hMGS-wt*). *Elav-Gal4*^C155^ was used as a control. The flies were cultured at the density of ∼100 flies in 40 ml cornmeal–agar medium at 25±1°C under a constant light-dark cycle with 70-80% humidity. Following eclosion, flies were aged for 3-4 days and transferred to fresh bottles with live yeast-containing media for serial cultures. Approximately 20 flies in 5 ml cornmeal–agar medium per 40 ml volume glass vials were employed for experiments.

For the heat shock experiments, age-matched (∼1-week old) flies for each genotype were anesthetized and ten flies, each in two or three replicates, for each gender (*N*=40-60) were transferred in media containing experimental glass vials. The flies were transferred back to the humidity chamber (25±1°C under a constant light-dark cycle; 70-80% humidity) and were allowed to recover for 24 h to avoid any anesthesia related effects on the treatment. On day 1, the flies were first pretreated at 30°C for 30 min and incubated at severe heat stress, 38°C for 1 h in a temperature-controlled incubator. Post-treatment, the number of flies paralyzed immediately after the treatment, and survival post-24-h recovery were calculated for all the genotypes. For the recovery analysis, the experimental vials were transferred back to the humidity chambers to allow for recovery at a constant temperature and humidity.

### Statistical analysis

All the results were presented as the mean of three independent experiments. Error bars were calculated by mean±s.e. The data were tested using an unpaired Student's *t*-test in GraphPad software. A *P*-value of ≤0.05 was considered statistically significant. Bar diagrams were prepared by using prism software.

## Supplementary Material

10.1242/biolopen.061605_sup1 Supplementary information
