# Supplementary material for: Glycogen synthase is required for heat shock-mediated autophagy induction in neuronal cells
Source: Biol Open. 2025 Feb 17;14(2):BIO061605. doi: 10.1242/bio.061605 (PMC11876841; doi:10.1242/bio.061605)
Supplement: Supplementary information [file biolopen-14-061605-s1.pdf]

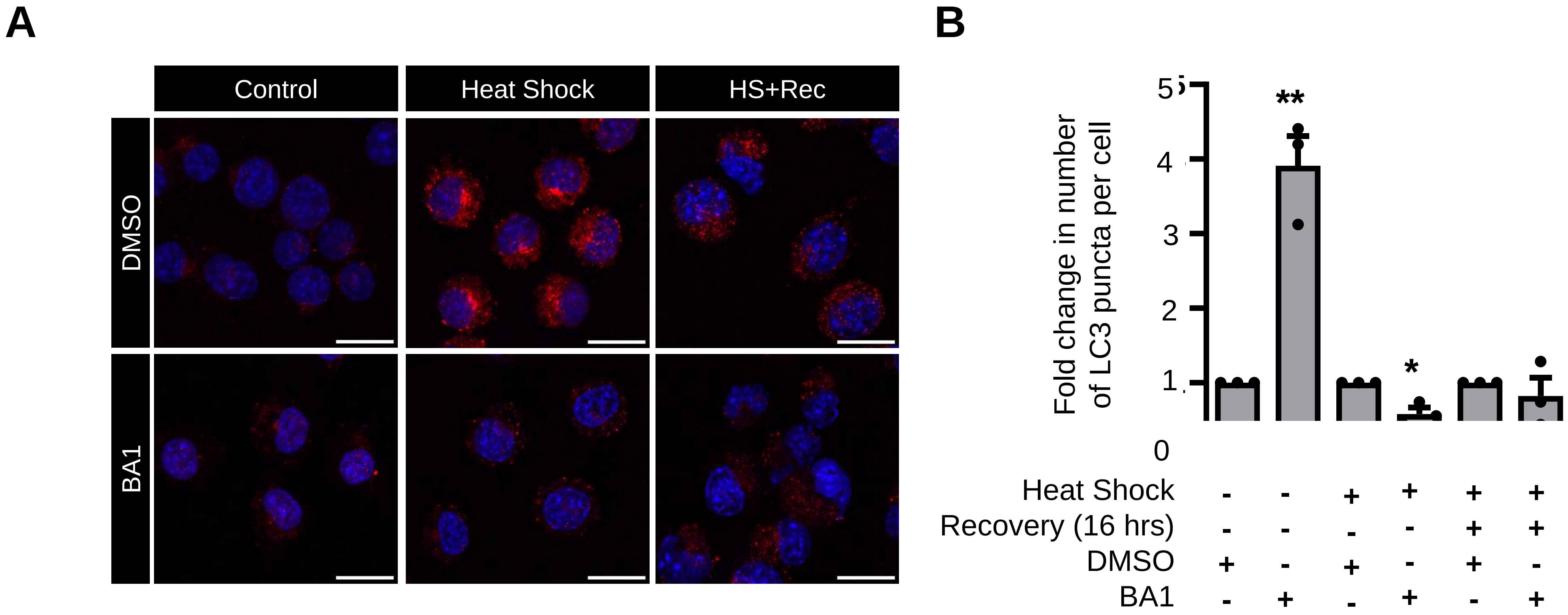

Fig. S1. Representative immunofluorescence images (A) showing the endogenous level of total LC3 protein (red puncta) and bar diagram (B) showing the fold change in the no. of LC3 puncta per cell in control, heat shock and recovery periods with and without bafilomycin treatment (35-40 cells each set). The nuclei in (A) were stained with DAPI (Scale bar, 20 µm). Data shown in are represented as mean ±s.e.; (n=3); *, $p<0.05$; **, $p<0.01$

Biology Open • Supplementary information

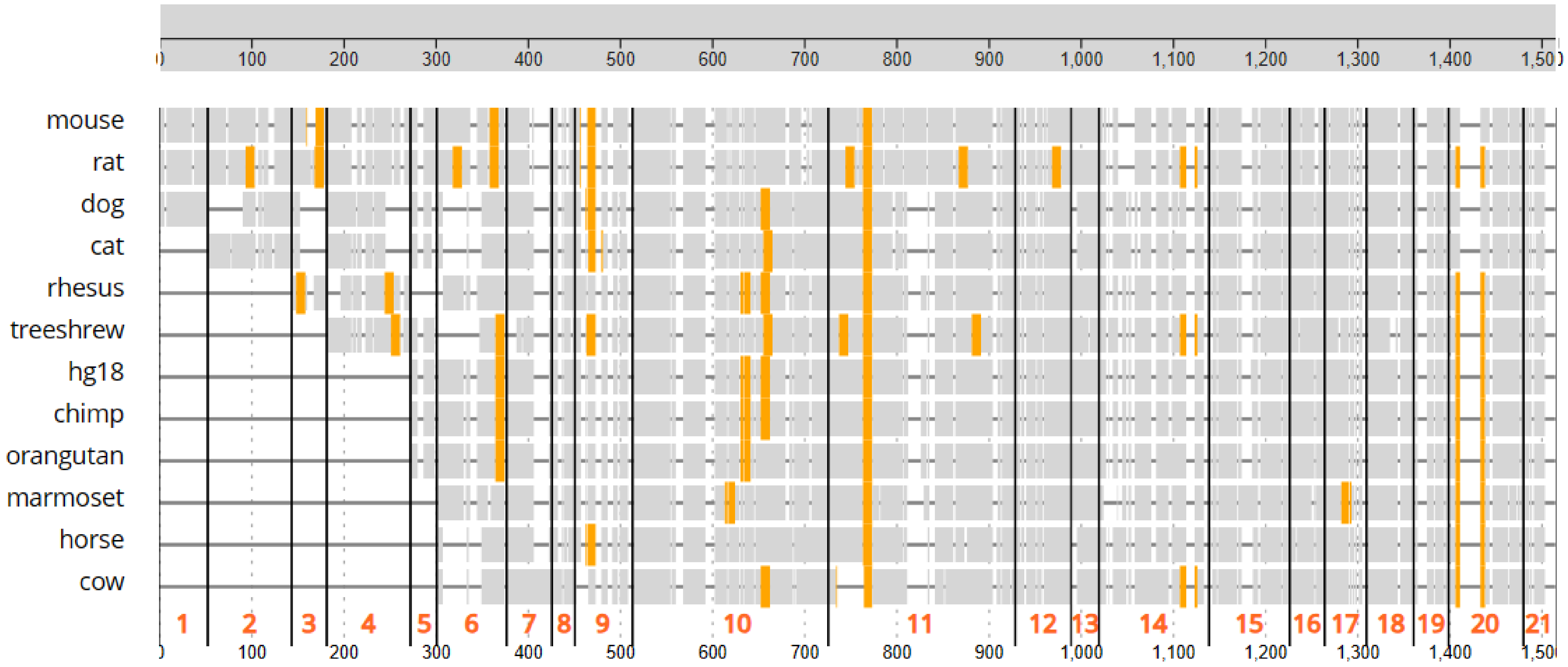

Fig. S2. Figure showing that the HSF1 binding motifs are conserved in GYS1 promoter sequence: Schematic diagram showing that HSF1 binding sequences (highlight in yellow colour) in GS promoter region by using the ConTra v3 tool. We find HSF1 binding sites in the 1kb region upstream to the transcription start site and 5'UTR of GYS1 gene in mice. The region between 700-800bp upstream is particularly noteworthy as it is conserved across the species mentioned.

Biology Open • Supplementary information

Table S1. Table showing primer sequences used for the RT-PCR in this study

Name of the gene	Primer Sequence (FWD- Forward primer, RVS- reverse primer)	Annealing Temperature
GYS1	FWD- 5’GTGACAGGGGATGAATGGGG-3’<br>RVS-5’-AACCCATGACTCCAGACACC-3’	60
HSP70	FWD-5’TGGTGCTGACGAAGATGAAG-3’<br>RVS-5’AGGTCGAAGATGAGCACGTT-3’	60
GAPDH	FWD-5’-AAGGTCATCCCAGAGCTGAA-3’<br>RVS-5’-CTGCTTCACCACCTTCTTGA-3’	60
